# Effects of Pistachio Consumption in a Behavioral Weight Loss Intervention on Weight Change, Cardiometabolic Factors, and Dietary Intake

**DOI:** 10.3390/nu12072155

**Published:** 2020-07-20

**Authors:** Cheryl L. Rock, Elizabeth Zunshine, Huong Thien Nguyen, Annemarie O. Perez, Christine Zoumas, Bilge Pakiz, Martha M. White

**Affiliations:** Department of Family Medicine and Public Health, School of Medicine, University of California, San Diego, La Jolla, CA 92093-0901, USA; elquintana@ucsd.edu (E.Z.); htnguyen@ucsd.edu (H.T.N.); aop001@ucsd.edu (A.O.P.); czoumasmorse@ucsd.edu (C.Z.); bpakiz@ucsd.edu (B.P.); mmwhite@ucsd.edu (M.M.W.)

**Keywords:** pistachios, weight loss, nuts, dietary intake, Healthy Eating Index, cardiometabolic risk factors, blood pressure

## Abstract

Epidemiological studies have linked regular nut consumption with lower body mass index and reduced likelihood of weight gain in adulthood. Nuts can displace other foods in the diet, and thus, promote a healthier dietary pattern. The purpose of this study was to examine the effect of pistachio nut consumption in overweight/obese adults. This randomized controlled study enrolled non-diabetic overweight/obese adults (*n* = 100) assigned to a 4-month behavioral weight loss intervention only group (controls) or also prescribed 1.5 oz/day (42 g/day) of pistachios (pistachio group). Outcomes were change in body weight, cardiometabolic factors, and dietary intake. Percent weight change was similar in the two groups (−5.1 [0.5] (mean [SE])% in the control group and −4.9 [0.6]% in the pistachio group, and body mass index (BMI) and waist circumference were reduced in both groups (time effect *p* ≤ 0.05). The pistachio group (but not the control group) exhibited a significant reduction in both systolic and diastolic blood pressure (time effect *p* = 0.01). Plasma alpha-carotene, beta-carotene, and lutein concentrations increased significantly in the pistachio group (time effect *p* < 0.05). Pistachio consumption was associated with increased dietary fiber intake and decreased consumption of sweets. Regular consumption of pistachios was associated with a comparable degree of weight loss, and similar reductions in BMI and waist circumference, in overweight/obese men and women compared to controls, and favorable changes in the diet, in the context of a behavioral weight loss intervention.

## 1. Introduction 

Nuts are energy-dense but also nutrient-dense, high in protein, fiber and micronutrients, and low in saturated fatty acids, and thus are part of a healthy dietary pattern [1]. Epidemiological studies have linked regular consumption of nuts with lower body mass index (BMI) and reduced likelihood of weight gain in adulthood [2]. In a meta-analysis of two large prospective cohort studies, the relative risk for every serving per week of nuts was 0.97 (95% CI: 0.95, 0.98) for overweight/obesity and 0.95 (95% CI: 0.89, 1.02) for obesity [3]. Analysis of pooled data from randomized feeding studies indicates that a nut-enriched diet, compared with the control diet, was associated with a significant reduction in body weight in 56 studies (−0.22 kg, 95% CI: −0.44, −0.04 kg), BMI in 39 studies (−0.16 kg/m^2^, 95% CI: −0.31, −0.01 kg/m^2^), and waist circumference in 23 studies (−0.51 cm, 95% CI: −0.95, −0.07 cm) [3]. Several clinical trials and epidemiological studies have also linked regular nut consumption with lower levels of cardiometabolic risk factors and risk for cardiovascular disease [4,5].

Notably, the largest proportion of the nut feeding studies and clinical trials reported to date have tested the effects of almonds and walnuts, with fewer studies testing the effects of other nuts, and there are some differences in levels of nutrients and biologically active constituents across the different types of nuts. Pistachios contain the highest levels of gamma-tocopherol, vitamin K, potassium, phytosterols, beta-carotene, and lutein, compared to other nuts [6,7]. The effects of prescribing pistachios on body weight and dietary intake of free-living subjects has been examined in only a few previous studies [8,9,10,11,12], and only one of these studies was in the context of a weight loss intervention specifically targeting overweight and obese individuals [8]. 

Several mechanisms have been suggested to explain why nut consumption does not promote weight gain that would be expected in clinical studies and is associated with reduced risk of obesity [13,14]. Nuts contribute less metabolizable energy in the human biological system than is calculated by proximate analysis and standardized Atwater estimates [15,16,17], and the polyunsaturated fatty acids in nuts may increase postprandial thermogenesis [18]. Nuts can increase satiety and also can promote dietary compensation or displacement of other foods, resulting in diet modifications [19]. Individuals who regularly consume nuts have been observed to have different dietary consumption patterns when compared to those who do not consume nuts [20,21]. 

The specific aims of this study were: To examine the effects of prescribing a pistachio-enriched reduced-energy diet on weight change compared to general dietary guidance and strategies in a 4-month behavioral weight loss intervention; to examine whether there is a differential response in metabolic and cardiovascular disease risk factors in association with a pistachio-enriched diet, and to examine whether there is a change in dietary intake and food choices in response to being prescribed a pistachio-enriched diet. These aims were addressed in a randomized controlled study involving non-diabetic overweight or obese men and women participating in a behavioral weight loss intervention. We hypothesized that participants assigned to the pistachio group would have comparable or greater weight loss and improvements in metabolic and cardiovascular disease risk factors compared to controls. We also hypothesized that dietary intake in the pistachio group would differ from that of participants receiving the behavioral intervention with general dietary guidance and strategies (the control group).

## 2. Materials and Methods

### 2.1. Subjects

**Figure 1:**
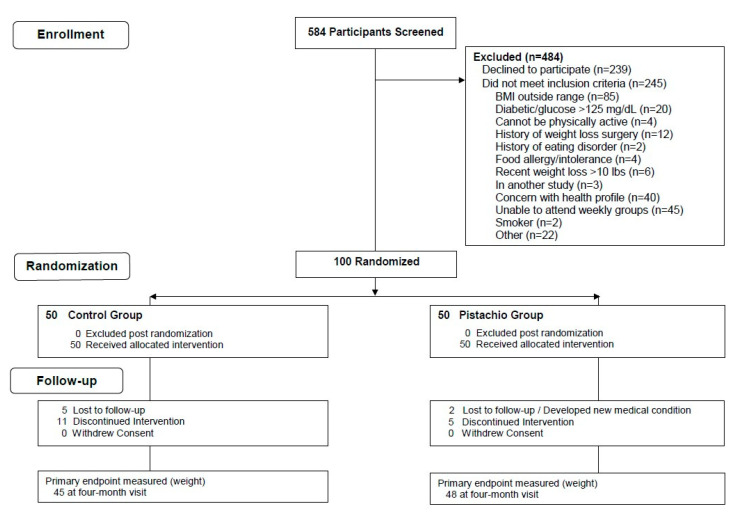
Flow chart for study participants.

Overweight or obese men and women were recruited from the community through mailings and email blasts and were prescreened using a telephone interview, followed by a screening and data collection clinic visit. One hundred individuals were randomized from a screened sample of 584 (Figure 1). Inclusion criteria were: Aged 21 years and older, BMI between 27–40 kg/m^2^; willing and able to participate in clinic visits, group sessions, and telephone and internet communications; able to provide data through questionnaires and telephone; willing to maintain contact with the investigators for four months; willing to allow blood collections; no known allergy to tree nuts; non-smoker; and capable of performing a simple test for assessing cardiopulmonary fitness. Exclusion criteria were: Inability to participate in physical activity due to severe disability; history or presence of a comorbid disease for which diet modification and increased physical activity may be contraindicated; self-reported pregnancy or breastfeeding or planning a pregnancy within the next year; currently involved in another diet intervention study or weight loss program; having another member of the household participating in the study; and having a history or presence of a significant psychiatric disorder or any condition that would interfere with participation in the trial.

Prior to enrollment and randomization, subjects were screened for diabetes and considered ineligible with a fasting blood glucose ≥125 mg/dL. Once enrolled, participants were randomly assigned to one of the two study arms using a sequence stratified by age (≤52 vs. >52 years) and BMI (≤33 vs. >33 kg/m^2^).

The study protocol and consent form were approved by the University of California, San Diego (UCSD), Institutional Review Board (number 172109). All participants provided written informed consent. The trial is registered at http://www.clinicaltrials.gov (NCT03387709).

### 2.2. Intervention

The group-based behavioral weight loss intervention, which was provided to all study participants, consisted of several elements of cognitive-behavioral therapy for obesity. Briefly, strategies and approaches that are applied in this type of intervention include: Self-monitoring of dietary intake and physical activity (via Internet, telephone app, and print-logbook options); goal-setting, using behavior-specific goals and a step-wise approach to promote self-efficacy; stimulus control and environmental management; training in problem-solving; and relapse prevention as a specific application of cognitive restructuring. Participants were advised to reduce energy intake by 500–1000 kcal/day below estimated maintenance requirements and to aim for a weight loss of 1–2 pounds/week. The physical activity component emphasized planned aerobic exercise, increased physical activity in the lifestyle, and strength training. The long-term goal was to achieve an average of 60 min/day of planned aerobic exercise at a moderate or strenuous level of intensity, which is consistent with the current recommendations for weight management [1,22]. During the 4-month intervention, all study participants had weekly email and text message contacts to provide support and behavioral guidance and strategies. 

The pistachios provided to participants assigned to that study group were roasted, shelled and unsalted kernels (American Pistachio Growers, Fresno, CA, USA) packaged individually into 1.5-oz. portions. The goal was to consume approximately 1.5 oz/day (42 g/day) or 18% of energy intake. Participants assigned to the pistachio group had the same overall goal of achieving a deficit of 500–1000 kcal/day, as communicated in the behavioral weight loss intervention, but in an initial counseling session with a dietitian, they were provided recipes and examples of how they might choose to include the nuts in meals and snacks. They were instructed to store them in the refrigerator or a cool place. The pistachios were distributed every 3–4 weeks, and compliance was assessed via a self-monitoring form that participants completed and submitted each time they received a new supply of pistachios.

Participants not randomized to the pistachio group were requested to abstain from consuming nuts during the study. All participants had contact with the project coordinator a minimum of every 1–2 weeks throughout the period of their involvement in the study, with the aim of equalizing contact with research staff in the two study groups.

### 2.3. Measurements

#### 2.3.1. Clinical and Anthropometric Measures

At scheduled clinic visits (baseline and four months), weight, height, and waist circumference were measured, and a blood sample was collected. Weight was obtained with participants in light clothing and using a calibrated Tanita digital scale. Waist circumference was measured by taking measures directly on the skin (displacing any clothing), with the abdomen relaxed and upon exhale and feet together, using a non-stretch, tension controlled Gulick measuring tape. Participants were confirmed to have been fasting at least 12 h with only water hydration allowed prior to drawing blood. Venous blood was collected into SST and heparin vacutainers using standard venipuncture procedures by certified phlebotomists. Processing for serum and plasma aliquots was performed at room temperature in low-light conditions or under gold lights to reduce degradation of carotenoids, and cryovials were stored in −80 °C freezers until analysis.

Prior to the visit, participants were mailed study questionnaires to be completed and brought to the clinic visit, and the study coordinator reviewed these forms for valid responses and completeness. In addition, two systolic and diastolic blood pressure measurements were made, using a standard automated device, and the average was used in analysis and reporting.

A 3-min stepping test was used to assess cardiopulmonary fitness of participants at both clinic visits, which was paced and standardized using a metronome. This test measures heart rate by taking the pulse for 30 s immediately after a 15 s recovery period from stepping. Step tests are useful for field-testing subjects, and although they are less accurate than measuring maximal oxygen uptake (VO_2max_), they have high reliability and are sensitive to change [23]. 

#### 2.3.2. Dietary Assessment

Detailed dietary data were collected from all study participants at three time points (baseline, two months, and four months) to assess and compare dietary intake over time and to examine food choices as an evaluation of displacement (i.e., compensatory responses to regular pistachio consumption) or diet modification. 

Dietary intake data were collected and analyzed using Nutrition Data System for Research software version NDSR 2017 developed by the Nutrition Coordinating Center (NCC), University of Minnesota, Minneapolis, MN, USA [24]. The NDSR software utilizes the multiple-pass system to improve recall accuracy, to prompt assessors to obtain detailed data about preparation methods and the type and amount of food eaten, and to guide and standardize data entry. Twenty-four h dietary recalls were conducted by telephone in sets of three recalls (2 weekdays and 1 weekend day) by trained dietary assessors. The baseline 24 h recalls were collected within the week of the initial clinic visit, and the subsequent two sets of 24 h recalls were collected during a 2-week period within two to four months following randomization. 

Diet quality was assessed using the Healthy Eating Index 2015 (HEI-2015), which is a tool assessing diet quality as specified by the 2015 Dietary Guidelines for Americans [1] using NDSR software. Possible index points range from 1–100, with a higher score indicating greater consistency of the diet with the guidelines and includes 13 dietary components (nine adequacy and four moderation components) that reflect diet quality. The NDSR HEI-2015 software program was written to generate the needed measures and derive scores for each index component in accord with the HEI-2015 scoring system.

#### 2.3.3. Laboratory Measurements 

For the lipid panel, glucose and insulin quantification, a minimum of 0.3 mL of serum was submitted for each participant (baseline and follow-up) to the University of Vermont Laboratory for Clinical Biochemistry Research (Burlington, VT, USA) for testing. The lipid panel (triglycerides, total cholesterol, and high-density lipoprotein [HDL] cholesterol) was measured by enzymatic colorimetric test and glucose was measured by UV photometry using a Roche Cobas C311 analyzer. LDL cholesterol was calculated from the total and HDL cholesterol values using the Friedewald equation [25]. Insulin was measured using electrochemiluminescence on the Roche Cobas e411. Homeostasis model assessment-insulin resistance (HOMA-IR) was computed from the measured levels of insulin and glucose, ([fasting glucose, mmol/L] × [insulin, mIU /L]/22.5) with HOMA-IR >3.0 considered indicative of insulin resistance. This approach to assess insulin resistance status has been validated in previous studies and is considered an acceptable indicator of insulin resistance [26]. The coefficient of variation (CV) for human serum for cholesterol is 2.4%; for triglycerides 3.5%; and for HDL cholesterol <0.1%. The CV for insulin is 3.7% and for glucose is 1.8%.

Plasma carotenoids were separated and quantified using high performance liquid chromatography (HPLC), and the extraction of carotenoids and separation methods have been previously described [27]. Zeaxanthin and lutein elute together with this analytical method, which quantifies >90% of the carotenoids present in the circulation in humans. The HPLC system (Varian ProStar/Star Workstation, Agilent 1100/Chemstation) measures the carotenoid panel (alpha-carotene, beta-carotene, lycopene, lutein, and beta-cryptoxanthin) using reversed phase separation and visible photometry on the Varian Prostar and Agilent 1100 HPLC system, with comparable results validated by quality controls. Separation was achieved using a Supelcosil LC-18 column (Supelco, 58,298; 25 cm × 4.6 mm, 5 µm particle size) and gradient elution.

#### 2.3.4. Other Measurements and Questionnaires

Physical activity was estimated with the validated Godin Leisure-Time Exercise Questionnaire which allows an assessment of weekly hours of moderate and strenuous physical activity [28]. The SF-36 Quality of Life (QOL) Questionnaire, which is a general measure of physical and mental QOL, was administered at baseline and four months. It is comprised of an 8-scale profile of functional health and well-being scores as well as psychometrically-based physical and mental health summary measures [29]. At baseline and 4-month clinic visits, participants also completed the three-factor Eating Inventory, a 51-item questionnaire that assesses eating attitudes and behavior across three scales: dietary restraint, disinhibition, and hunger, as well as subscales of these constructs [30,31,32,33]. 

### 2.4. Statistical Analysis

A Research Electronic Data Capture (REDcap) database hosted at UCSD was used to collect and manage all study data [34]. QOL scores were calculated as recommended by RAND Health Care. HEI-2015 scores were calculated using SAS code for data collected in NDSR 2017.

Analyses were conducted using SAS version 9.4 (SAS Institute, Inc., Cary, N.C., USA). Comparisons were made between the study arms at each time interval, and between baseline and follow-up values within each study arm. All tests were two-tailed with a significance level of α = 0.05. The demographic variables for sex and race/ethnicity were tested using chi-square tests. For all other parameters, group means were compared using t-tests, with the Satterthwaite method for unequal variances. Power for this sample size was estimated based on previously published literature on nut consumption in a weight loss intervention [8,35,36]. To detect a 3% (SD = 5%) difference in body weight at four months between groups, there is 80% power with 45 subjects per group. Thus, allowing for 10% drop-out or loss to follow-up at four months, we recruited a total of 100 participants in the study.

## 3. Results

A total of 100 adults were enrolled in this study, and 50 were randomized to each of the two study groups (Figure 1). Approximately two-thirds of the participants were women, and gender distribution was similar in the two study groups (Table 1). The average age was 55.6 years, and the average BMI was 32.8 kg/m^2^. Six participants dropped out before completing the study, and one developed a new medical condition (unrelated to study participation) during the course of the study. Eighty-four percent of the subjects completed the full four months of the weekly behavioral weight loss intervention group meetings (Figure 1). One participant from each study group declined the final blood draw. Not all participants completed dietary recalls at two and four months, but the majority completed the recalls and the rate of completion was similar in the two study groups. Compliance with pistachio intake among those assigned to the pistachio group was very good overall: 41 reported consuming >80%, 4 reported consuming 70–79%, and 5 reported consuming <70% of the prescribed pistachios. There were no serious adverse events.

There were no significant differences in body weight, BMI, and waist circumference at baseline between the two groups (Table 2). Percent weight change at study end was similar in the two groups (−5.1 [0.5] (mean [SE])% in the behavioral intervention only group and −4.9 [0.6]% in the pistachio-enriched diet group), and the change did not reach statistical significance. BMI and waist circumference were reduced at four months in both groups (time effect *p* ≤ 0.05) (Table 2). At study end, the reduction in body weight, BMI, and waist circumference at four months did not differ between the groups.

The pistachio group (but not the control group) exhibited a significant reduction in both systolic and diastolic blood pressure (time effect *p* = 0.01), although a significant difference between the groups was not observed at either time point (Table 3). Both groups significantly increased moderate/strenuous physical activity levels (time effect *p* < 0.01), approximately doubling their minutes/week of moderate/strenuous physical activity. Both groups also exhibited a significant improvement in cardiopulmonary fitness as assessed by the step test (time effect *p* < 0.02) (Table 3).

Although total cholesterol and triglyceride levels decreased in both groups, the reduction did not reach statistical significance (Table 4). HDL cholesterol levels did not change significantly over the course of the study in either group, and LDL cholesterol levels declined in both groups but did not reach statistical significance. Baseline and 4-month insulin and glucose levels were similar across the groups and did not change over the course of the study, so HOMA-IR was similar in both groups at both time points (Table 4). The pistachio group exhibited significant increases in alpha-carotene, beta-carotene, and lutein concentrations at study end (time effect *p* < 0.05). Among the carotenoids, only lutein increased in the control group (time effect *p* = 0.05).

Energy intake was similar at baseline in the study groups and declined significantly by study end in both groups (time effect *p* < 0.001), with no significant differences between them at 2- and 4-month follow-up time points (Table 5). Percent energy from fat was significantly higher in the pistachio group than the control group at both 2- and 4-month follow-up time points (between groups *p* < 0.001 and *p* = 0.05, respectively). Fiber intake significantly increased in the pistachio group at two months (time effect *p* < 0.01) but did not reach significance at four months, while fiber intake did not change significantly in the control group at either follow-up time point. 

Analysis of food group consumption revealed a few differences in intakes between the study groups. The pistachio group reported a higher intake of high-protein foods (a category that includes nuts) than the control group at both follow-up time points (between groups *p* ≤ 0.05). The pistachio group reported a lower intake of sweets than the control group at four months (between groups *p* < 0.05), with the pistachio group reporting one-half the mean level of consumption of sweets as the controls. The pistachio group also reported a reduction in the intake of added fat (e.g., butter, margarine, oil, salad dressing) at both follow-up time points (time effect *p* < 0.05), which was not observed in the control group. 

The total HEI-2015 score improved significantly in both study groups at both the 2-and 4-month follow-up time points and did not differ between the groups (Table 5). The fatty acid ratio component, which reflects the ratio of poly- and monounsaturated fatty acids to saturated fatty acids, increased in the pistachio group at two months (time effect *p* < 0.001) and was significantly higher than the control group at both follow-up time points (between groups *p* < 0.05).

The Eating Inventory scores for dietary restraint increased and for disinhibition and hunger decreased significantly for both study groups at study end (time effect *p* < 0.001), and there was a trend in the pistachio group for lower disinhibition than the control group at study end (between groups *p* = 0.05). Subscale scores reflected the changes observed in the main factors and did not differ between the groups (data not shown). Significant changes in QOL scores for physical functioning, role limitations, and energy/fatigue were not observed in either group at study end.

## 4. Discussion

Regular consumption of pistachios was associated with a comparable degree of weight loss, and similar and significant reductions in BMI and waist circumference, in overweight/obese men and women in a behavioral weight loss intervention compared to controls who received only general dietary guidance. Additionally, pistachio consumption was associated with a reduction in both systolic and diastolic blood pressure, which was not observed in controls, despite substantial, similarly increased moderate/strenuous physical activity and cardiopulmonary fitness in both groups. Significant changes in lipids, glucose and insulin were not observed in the subjects prescribed pistachios or the controls. However, the study population was non-diabetic and generally had normal blood lipids, glucose and insulin at baseline, which reduces the likelihood of observing a reduction in these factors in response to pistachio intake or weight loss.

Regular pistachio consumption was associated with several shifts in dietary intake and food choices compared to controls, including increased dietary fiber and decreased consumption of sweets. The total HEI-2015 score increased in both study groups, and notably, those prescribed pistachios (but not controls) showed improvement in the fatty acid ratio component. 

Only a few randomized controlled clinical studies have examined the effects of prescribing pistachios on body weight and dietary intake in free-living study populations [8,9,10,11,12]. Findings from feeding studies in which pistachio-enriched and comparison diets are controlled and typically purposely isocaloric provide insight into potential effects on cardiometabolic factors but do not provide a relevant comparison for examining effects on body weight and dietary intake [37,38,39]. 

The most appropriate comparison of findings in the present study is a prior clinical trial of pistachios in a weight loss intervention with a similar sample (overweight and obese individuals) [8]. In that study, participants (*n* = 59) were prescribed an isocaloric reduced-energy diet that included an afternoon snack of 53 g pistachios or 56 g pretzels in a 12-week weight loss intervention [8]. There was a trend but not a significant difference in weight change in that study, although the pistachio group did exhibit a significantly greater reduction in BMI and plasma triglyceride concentration. The length of the intervention was relatively brief, similar to that of the present study (three vs. four months), which likely explains why weight change did not achieve statistical significance as we observed. Similar to the results of the present study, significant changes in total cholesterol and HDL and LDL cholesterol were not observed.

A recent randomized clinical trial examined the effects of a daily 56-g pistachio snack compared to an isoenergetic biscuit for four weeks on body weight, dietary intake and meal satiety in 60 healthy, normal weight (BMI 18.5–25 kg/m^2^) women [12]. The pistachio snack did not affect body weight or meal satiety in that study but was associated with higher intake of selected micronutrients. In a crossover study with 10-week treatment periods, prescribing 20% of energy from pistachios in 48 healthy, normal weight women was not associated with changes in body weight, blood lipids or blood pressure, although diet quality improved, including an increase in unsaturated fat and dietary fiber intake, similar to the observations in the present study [11]. 

Two previous clinical studies have examined effects of pistachios on body weight and cardiometabolic factors in sample populations of individuals with metabolic syndrome, with divergent results. In a randomized controlled study [9], 90 subjects with metabolic syndrome were instructed with the American Heart Association Step 1 Diet and assigned to consume 42 or 70 g/day pistachios or no pistachios for 12 weeks. In that study, there were no changes in body weight or waist-to-hip ratio in any group, and no differences across the groups in triglycerides, fasting glucose, 2 h postprandial glucose and blood pressure. In a similar randomized clinical trial, 60 subjects with metabolic syndrome were assigned to consume 20% of energy from pistachios (intervention group) vs. standard dietary guidelines, and the 24 week intervention was associated with statistically significant improvements in waist circumference and levels of fasting blood glucose, total cholesterol, LDL cholesterol, and inflammatory and oxidative stress factors [10]. Although a reduction in blood pressure was not observed in these previous clinical studies, this response was observed in a pistachio (20% of energy) crossover controlled feeding study of 30 adults with type 2 diabetes over a 4-week intervention [37].

Change in dietary intake and food choices in free-living individuals in response to prescribing other nuts (walnuts, almonds, and hazelnuts) has been examined in a few previous studies [19,40,41,42,43,44]. In most of these studies, displacement of other foods generally improved nutritional quality of the diet and may also explain why the addition of nuts to the diet does not promote weight gain.

Among the tree nuts, pistachios are notable for containing a relatively high level of lutein and also beta-carotene [6,7], but green and orange vegetables (and fruit) are also such good sources of these carotenoids that plasma levels are considered an excellent biomarker of fruit and vegetable intake [45]. In a controlled crossover feeding study, serum lutein increased significantly compared to a lower-fat control diet following four weeks of 1 serving/day (32–63 g/day) or 2 servings/day (63–126 g/day) of pistachios [38]. In that study, alpha-carotene and beta-carotene also increased in response to the pistachio-enriched diet, although the percent increase differed significantly from the control diet only at the higher dose of nuts. Similarly, these three carotenoids increased in the pistachio group in the present study, and the increased plasma lutein in the control group likely reflects an increased intake of green vegetables. 

This study has some limitations but also strengths. The weight loss intervention was relatively brief, so the amount of weight loss associated with a moderately energy-restricted diet was not substantial. The weight loss intervention was multifaceted, so attributing responses to specific behavioral changes and dietary factors is difficult. Additionally, we did not conduct biochemical measures of compliance and body composition. Although the study participants were overweight or obese, their blood levels of the measured cardiometabolic factors were generally normal at baseline, so their responses may not predict the responses in other higher-risk groups. Rather than testing across a range of pistachio consumption, we prescribed an amount achievable on a regular basis and compatible with typical food patterns, and it is possible that larger amounts may have different effects. The dietary intake data were self-reported and thus are subject to possible recall bias, as participants may not accurately and fully recall their intakes. However, the 24 h dietary recall method focuses only on the preceding day’s intake, rather than over a prolonged period of time. Strengths of this dietary assessment methodology are that the multiple-pass interview-based method can elicit detailed intake data, and NDSR provides a complete nutrient profile for all foods in the database. Other strengths of this study are the heterogeneity of the study sample, which included both men and women, and represented various racial/ethnic groups, a high retention rate that is not typical of weight loss studies, and the high compliance with the prescribed pistachios in that study group. 

## 5. Conclusions

Pistachios are a nutrient-dense tree nut that can contribute to a healthy dietary pattern and weight reduction, in the context of an energy-restricted diet in a behavioral intervention, and may confer additional health benefits such as a reduction in systolic and diastolic blood pressure. Additionally, regular pistachio consumption is associated with healthful shifts in dietary intake and food choices, including increased dietary fiber, decreased consumption of sweets, and a more favorable ratio of poly-and monounsaturated fatty acids to saturated fatty acids.

## Figures and Tables

**Table 1:** Characteristics of study participants in the weight loss intervention ^1^.

Variable	Control Group *n* = 50	Pistachio Group *n* = 50
Sex ^2^		
Female	32 (64)	30 (60)
Male	18 (36)	20 (40)
Age (years) ^3^	56.2 (1.5)	55.0 (1.6)
Education (years) ^3^	16.4 (0.3)	16.4 (0.3)
Race/ethnicity (%)		
Non-Hispanic white	66	80
Hispanic/Latino	6	8
African American	8	4
Asian-American	4	4
Mixed/other	16	4

^1^ No significant differences between the groups based on chi-square tests. (categorical variables), or *t*-tests (continuous variables). ^2^ Mean (%). ^3^ Mean (SE).

**Table 2:** Body measurements of study participants in the weight loss intervention.

Variable	Baseline		4 Months	
	Control Group *n* = 50	Pistachio Group *n* = 50	Control Group *n* = 47	Pistachio Group *n* = 49
Body weight (kg)	93.8 (2.2) ^1^	94.7 (2.3)	88.9 (2.1)	90.0 (2.2)
Body mass index (kg/m^2^) ^2^	32.8 (0.5)	32.8 (0.6)	31.1 (0.5)	31.2 (0.6)
Weight change (kg)			−4.8 (0.5)	−4.7 (0.7)
% Weight change			−5.1 (0.5)	−4.9 (0.6)
Waist circumference (cm) ^2^	108.6 (1.6)	108.4 (1.4)	102.7 (1.6)	103.4 (1.6)

^1^ Mean (SE). ^2^ Body mass index and waist circumference showed a significant time effect compared with baseline, *p* ≤ 0.05 for each variable, in both study groups at four months.

**Table 3:** Blood pressure and physical activity variables for study participants in the weight loss intervention.

Variable	Baseline		4 Months	
	Control Group *n* = 50	Pistachio Group *n* = 50	Control Group *n* = 45	Pistachio Group *n* = 48
Systolic blood pressure (mm Hg)	124 (2) ^1^	130 (2)	121 (2)	121 (2) ^2^
Diastolic blood pressure (mm Hg)	81 (1)	83 (1)	77 (2)	78 (1) ^2^
Moderate/strenuous physical activity (minutes/week)	190 (30)	172 (22)	373 (62) ^2^	306 (29) ^3^
Step test (heart rate/30 s)	54 (1)	55 (1)	49 (1) ^2^	50 (1) ^2^

^1^ Mean (SE). ^2^ Different from baseline within group, *p* < 0.05 for each. ^3^ Different from baseline within group, *p* < 0.001.

**Table 4:** Biological measurements of study participants in the weight loss intervention.

Variable	Baseline		4 Months	
	Control Group *n* = 50	Pistachio Group *n* = 50	Control Group *n* = 44	Pistachio Group *n* = 47
Cholesterol (mg/dL)	206 (5) ^1^	204 (6)	194 (5)	194 (6)
Triglycerides (mg/dL)	123 (8)	135 (12)	108 (6)	123 (8)
HDL Cholesterol (mg/dL)	60 (2)	58 (2)	60 (2)	58 (2)
LDL Cholesterol (mg/dL)	121 (4)	119 (5)	112 (4)	111 (5)
Insulin (umol/mL)	14 (1)	14 (1)	13 (1)	13 (1)
Glucose (mg/dL)	98 (1)	99 (1)	99 (1)	99 (1)
Homeostasis model assessment-insulin resistance	3.4 (0.2)	3.4 (0.3)	3.3 (0.3)	3.1 (0.2)
Alpha-Carotene (umol/L)	0.22 (0.04) ^2^	0.12 (0.01) ^2^	0.60 (0.31)	0.24 (0.04) ^3^
Beta-Carotene (umol/L)	0.72 (0.13) ^2^	0.42 (0.04) ^2^	0.92 (0.12) ^2^	0.63 (0.08) ^2,3^
B-Cryptoxanthin (umol/L)	0.25 (0.03)	0.22 (0.03)	0.25 (0.04)	0.22 (0.04)
Lutein/Zeaxanthin (umol/L)	0.60 (0.05)	0.54 (0.05)	0.78 (0.07) ^2,3^	0.86 (0.10) ^2,3^
Lycopene (umol/L)	0.75 (0.06)	0.73 (0.06)	0.71 (0.06)	0.70 (0.06)

^1^ Mean (SE). ^2^ Different between groups, *p* ≤ 0.05. ^3^ Different from baseline within group, *p* ≤ 0.05.

**Table 5:** Dietary intake of study participants in the weight loss intervention.

Variable	Baseline		2 Months		4 months	
	Control *n* = 50	Pistachio *n* = 50	Control *n* = 41	Pistachio *n* = 46	Control *n* = 43	Pistachio *n* = 44
Energy (kcal/day)	1933 (79) ^1^	1843 (81)	1617 (106) ^2^	1649 (70)	1464 (62) ^3^	1488 (60) ^3^
% Energy from carbohydrate	42.5 (0.9)	40.7 (1.2)	42.3 (1.2)	39.5 (1.3)	43.9 (1.3)	41.7 (1.3)
% Energy from protein	18.5 (0.6)	18.5 (0.8)	21.2 (1.0) ^2^	19.0 (0.7)	20.2 (0.7)	19.7 (0.8)
% Energy from fat	35.7 (0.8)	37.7 (1.2)	33.9 (0.8) ^5^	38.7 (1.3) ^5^	33.7 (1.0) ^4^	36.6 (0.9) ^4^
Carbohydrate (g/day)	210.1 (10.5)	193 (10.1)	177.4 (11.6) ^2^	169.5 (9.5)	168.8 (9.1) ^2^	159.1 (7.8) ^2^
Protein (g/day)	89.9 (4.8)	82.7 (4.0)	85.8 (7.8)	78.5 (3.8)	74.4 (4.0) ^2^	74.4 (3.8)
Fat (g/day)	78.1 (3.3)	79.8 (4.6)	62.2 (4.5) ^2^	73.1 (3.8)	56.3 (2.7) ^3^	62.7 (3.1) ^2^
Dietary Fiber (g/day)	20.9 (1.2)	18.6 (0.9)	22.6 (1.8)	23.5 (1.4) ^2^	22.4 (1.6)	21.5 (1.3)
Fruit (avg servings/day)	1.4 (0.2)	1.3 (0.2)	2.3 (0.3) ^2^	1.7 (0.2)	1.8 (0.2)	1.6 (0.2)
Vegetables (avg servings/day)	3.9 (0.4)	3.4 (0.3)	4.4 (0.4)	4.1 (0.4)	4.4 (0.4)	4.0 (0.4)
Grains (avg servings/day)	6.1 (0.3)	5.4 (0.4)	4.3 (0.4) ^3^	4.1 (0.4) ^2^	4.3 (0.3) ^3^	4.3 (0.3) ^2^
Dairy/nondairy alternatives (avg servings/day)	1.5 (0.1)	1.5 (0.1)	1.4 (0.2)	1.2 (0.1) ^2^	1.4 (0.2)	1.2 (0.2)
Meat, fish, poultry, eggs, nuts, seeds (avg servings/day)	7.3 (0.6)	6.8 (0.4)	6.4 (0.6) ^4^	8.0 (0.5) ^4^	5.6 (0.5) ^2,4^	7.1 (0.5) ^4^
Sweets (avg servings/day)	0.9 (0.2)	0.9 (0.2)	0.9 (0.2)	1.2 (0.5)	1.0 (0.1) ^4^	0.5 (0.1) ^2,4^
Miscellaneous foods (avg servings/day)	1.5 (0.3)	1.4 (0.2)	1.6 (0.4)	1.4 (0.2)	1.2 (0.2)	1.5 (0.2)
Butter, margarine, oil, salad dressing (added fats) (avg servings/day)	4.2 (0.4)	4.3 (0.3)	3.4 (0.4)	3.2 (0.4) ^2^	3.2 (0.3)	2.9 (0.3) ^2^
Total HEI-2015 Score ^6^	63.5 (1.9)	60.9 (1.8)	69.1 (1.6) ^2^	72.5 (1.8) ^3^	69.0 (2.0) ^2^	70.3 (1.9) ^3^
HEI Component 1: Total vegetables	3.7 (0.2)	3.7 (0.2)	4.4 (0.2) ^2^	4.0 (0.2)	4.4 (0.2) ^2^	4.1 (0.2)
HEI Component 2: Greens and beans	3.6 (0.3)	3.1 (0.3)	4.2 (0.2) ^2^	4.0 (0.3) ^2^	4.3 (0.2) ^2,4^	3.4 (0.3) ^4^
HEI Component 3: Total fruit	2.1 (0.2)	2.0 (0.3)	3.4 (0.2) ^3^	2.8 (0.3) ^2^	3.2 (0.3) ^2^	2.9 (0.3) ^2^
HEI Component 4: Whole fruit	3.0 (0.3)	2.5 (0.3)	3.9 (0.3) ^2^	3.5 (0.3) ^2^	3.8 (0.3)	3.5 (0.3) ^2^
HEI Component 5: Whole grains	5.5 (0.5)	4.5 (0.5)	5.3 (0.6)	4.9 (0.6)	5.6 (0.5)	6.3 (0.5) ^2^
HEI Component 6: Dairy	5.3 (0.4)	5.3 (0.4)	5.4 (0.5)	4.8 (0.4)	5.9 (0.5)	5.0 (0.4)
HEI Component 7: Total protein foods	4.8 (0.1)	4.9 (0.1)	4.9 (0.1)	5.0 (0.0)	4.8 (0.1)	4.8 (0.1)
HEI Component 8: Seafood and plant protein	3.6 (0.3)	3.9 (0.2)	3.6 (0.3) ^5^	5.0 (0.0) ^3,5^	3.9 (0.3)	4.5 (0.2) ^2^
HEI Component 9: Fatty acid ratio	5.8 (0.5)	5.6 (0.5)	4.7 (0.5) ^5^	8.2 (0.4) ^3,5^	5.0 (0.5) ^4^	6.9 (0.5) ^4^
HEI Component 10: Sodium	4.8 (0.4)	4.8 (0.4)	5.5 (0.5)	5.7 (0.5)	4.6 (0.5)	5.5 (0.5)
HEI Component 11: Refined grains	6.8 (0.4)	6.7 (0.5)	8.5 (0.4) ^2^	8.2 (0.4) ^2^	7.9 (0.4) ^2^	7.3 (0.5)
HEI Component 12: Added sugars:	8.5 (0.3)	8.4 (0.3)	9.1 (0.3)	9.3 (0.2) ^2^	9.3 (0.2) ^2^	9.2 (0.2) ^2^
HEI Component 13: Saturated fats	6.1 (0.4)	5.4 (0.5)	7.4 (0.5) ^2^	7.3 (0.4) ^2^	7.3 (0.4) ^2^	7.4 (0.4) ^2^

^1^ Mean (SE). ^2^ Different from baseline within group, *p* ≤ 0.05 for each. ^3^ Different from baseline within group, *p* ≤ 0.001 for each. ^4^ Different between groups, *p ≤* 0.05 for each. ^5^ Different between groups, *p* < 0.001 for each. ^6^ Total Healthy Eating Index (HEI) 2015 Score.

## References

[1] U.S. Department of Agriculture, U.S. Department of Health and Human Services (2015–2020). Dietary Guidelines For Americans 2015–2020.

[2] Bes-Rastrollo M., Wedick N.M., Martinez-Gonzalez M.A., Li T.Y., Sampson L., Hu F.B. (2009). Prospective study of nut consumption, long-term weight change, and obesity risk in women. Am. J. Clin. Nutr..

[3] Li H., Li X., Yuan S., Jin Y., Lu J. (2018). Nut consumption and risk of metabolic syndrome and overweight/obesity: A meta-analysis of prospective cohort studies and randomized trials. Nutr. Metab..

[4] Kim Y., Keogh J., Clifton P.M. (2018). Nuts and cardio-metabolic disease: A review of meta-analyses. Nutrients.

[5] Del Gobbo L.C., Falk M.C., Feldman R., Lewis K., Mozaffarian D. (2015). Effects of tree nuts on blood lipids, apolipoproteins, and blood pressure: Systematic review, meta-analysis, and dose-response of 61 controlled intervention trials. Am. J. Clin. Nutr..

[6] Dreher M.L. (2012). Pistachio nuts: Composition and potential health benefits. Nutr. Rev..

[7] US Department of Agriculture A.R.S. (2016). USDA National Nutrient Database for Standard Reference.

[8] Li Z., Song R., Nguyen C., Zerlin A., Karp H., Naowamondhol K., Thames G., Gao K., Li L., Tseng C.H. (2010). Pistachio nuts reduce triglycerides and body weight by comparison to refined carbohydrate snack in obese subjects on a 12-week weight loss program. J. Am. Coll. Nutr..

[9] Wang X., Li Z., Liu Y., Lv X., Yang W. (2012). Effects of pistachios on body weight in Chinese subjects with metabolic syndrome. Nutr. J..

[10] Gulati S., Misra A., Pandey R.M., Bhatt S.P., Saluja S. (2014). Effects of pistachio nuts on body composition, metabolic, inflammatory and oxidative stress parameters in Asian Indians with metabolic syndrome: A 24-wk, randomized control trial. Nutrition.

[11] Burns-Whitmore B.B.A., Towne A.H., Roy S., Hall L.M. (2017). Pistachio consumption at 20% of energy does not significantly change body composition, blood pressure or blood lipids but improves diet quality in freeliving, healthy college-aged women. Food Nutr. J..

[12] Carughi A., Bellisle F., Dougkas A., Giboreau A., Feeney M.J., Higgs J. (2019). A randomized controlled pilot study to assess effects of a daily pistachio (pistacia vera) afternoon snack on next-meal energy intake, satiety, and anthropometry in french women. Nutrients.

[13] Tan S.-Y., Dhillon J., Mattes R. (2014). A review of the effects of nuts on appetite, food intake, metabolism, and body weight. Am. J. Clin. Nutr..

[14] Coe S. (2020). Nuts in the diet and bodyweight: What’s the relationship?. Nutr. Bull..

[15] Novotny J.A., Gebauer S.K., Baer D.J. (2012). Discrepancy between the Atwater factor predicted and empirically measured energy values of almonds in human diets. Am. J. Clin. Nutr..

[16] Baer D.J., Gebauer S.K., Novotny J.A. (2012). Measured energy value of pistachios in the human diet. Br. J. Nutr..

[17] Baer D.J., Gebauer S.K., Novotny J.A. (2016). Walnuts consumed by healthy adults provide less available energy than predicted by the Atwater factors. J. Nutr..

[18] Casas-Agustench P., Lopez-Uriarte P., Bullo M., Ros E., Gomez-Flores A., Salas-Salvado J. (2009). Acute effects of three high-fat meals with different fat saturations on energy expenditure, substrate oxidation and satiety. Clin. Nutr..

[19] Kranz S., Hill A.M., Fleming J.A., Hartman T.J., West S.G., Kris-Etherton P.M. (2014). Nutrient displacement associated with walnut supplementation in men. J. Hum. Nutr. Diet..

[20] O’Neil C.E., Keast D.R., Fulgoni V.L., Nicklas T.A. (2010). Tree nut consumption improves nutrient intake and diet quality in US adults: An analysis of National Health and Nutrition Examination Survey (NHANES) 1999–2004. Asia Pac. J. Clin. Nutr..

[21] O’Neil C.E., Nicklas T.A., Fulgoni V.L. (2015). Tree nut consumption is associated with better nutrient adequacy and diet quality in adults: National Health and Nutrition Examination Survey 2005–2010. Nutrients.

[22] (2014). Expert Panel: Report: Guidelines (2013) for managing overweight and obesity in adults. Obesity.

[23] McArdle W.D., Katch F.I., Katch V.L. (2007). Exercise Physiology: Energy, Ntrition, and Human Performance.

[24] Schakel S.F. (2001). Maintaining a nutrient database in a changing marketplace: Keeping pace with changing food products—A research perspective. J. Food Compos. Anal..

[25] Friedewald W.T., Levy R.I., Fredrickson D.S. (1972). Estimation of the concentration of low-density lipoprotein cholesterol in plasma, without use of the preparative ultracentrifuge. Clin. Chem..

[26] Bonora E., Targher G., Alberiche M., Bonadonna R.C., Saggiani F., Zenere M.B., Monauni T., Muggeo M. (2000). Homeostasis model assessment closely mirrors the glucose clamp technique in the assessment of insulin sensitivity: Studies in subjects with various degrees of glucose tolerance and insulin sensitivity. Diabetes Care.

[27] Rock C.L., Natarajan L., Pu M., Thomson C.A., Flatt S.W., Caan B.J., Gold E.B., Al-Delaimy W.K., Newman V.A., Hajek R.A. (2009). Longitudinal biological exposure to carotenoids is associated with breast cancer-free survival in the Women’s Healthy Eating and Living Study. Cancer Epidemiol. Prev. Biomark..

[28] Amireault S., Godin G., Lacombe J., Sabiston C.M. (2015). The use of the Godin-Shephard Leisure-Time Physical Activity Questionnaire in oncology research: A systematic review. BMC Med. Res. Methodol..

[29] Brazier J.E., Harper R., Jones N.M., O’Cathain A., Thomas K.J., Usherwood T., Westlake L. (1992). Validating the SF-36 health survey questionnaire: New outcome measure for primary care. BMJ.

[30] Hays N.P., Roberts S.B. (2008). Aspects of eating behaviors “disinhibition” and “restraint” are related to weight gain and BMI in women. Obesity.

[31] Westenhoefer J., Stunkard A.J., Pudel V. (1999). Validation of the flexible and rigid control dimensions of dietary restraint. Int. J. Eat. Disord..

[32] Bond M.J., McDowell A.J., Wilkinson J.Y. (2001). The measurement of dietary restraint, disinhibition and hunger: An examination of the factor structure of the Three Factor Eating Questionnaire (TFEQ). Int. J. Obes. Relat. Metab. Disord. J. Int. Assoc. Study Obes..

[33] Stunkard A.J., Messick S. (1985). The three-factor eating questionnaire to measure dietary restraint, disinhibition and hunger. J. Psychosom. Res..

[34] Harris P.A., Taylor R., Thielke R., Payne J., Gonzalez N., Conde J.G. (2009). Research electronic data capture (REDCap)—A metadata-driven methodology and workflow process for providing translational research informatics support. J. Biomed. Inform..

[35] Abazarfard Z., Salehi M., Keshavarzi S. (2014). The effect of almonds on anthropometric measurements and lipid profile in overweight and obese females in a weight reduction program: A randomized controlled clinical trial. J. Res. Med. Sci. Off. J. Isfahan Univ. Med. Sci..

[36] Foster G.D., Shantz K.L., Vander Veur S.S., Oliver T.L., Lent M.R., Virus A., Szapary P.O., Rader D.J., Zemel B.S., Gilden-Tsai A. (2012). A randomized trial of the effects of an almond-enriched, hypocaloric diet in the treatment of obesity. Am. J. Clin. Nutr..

[37] Sauder K.A., McCrea C.E., Ulbrecht J.S., Kris-Etherton P.M., West S.G. (2014). Pistachio nut consumption modifies systemic hemodynamics, increases heart rate variability, and reduces ambulatory blood pressure in well-controlled type 2 diabetes: A randomized trial. J. Am. Heart Assoc..

[38] Kay C.D., Gebauer S.K., West S.G., Kris-Etherton P.M. (2010). Pistachios increase serum antioxidants and lower serum oxidized-LDL in hypercholesterolemic adults. J. Nutr..

[39] Hernandez-Alonso P., Salas-Salvado J., Baldrich-Mora M., Juanola-Falgarona M., Bullo M. (2014). Beneficial effect of pistachio consumption on glucose metabolism, insulin resistance, inflammation, and related metabolic risk markers: A randomized clinical trial. Diabetes Care.

[40] Njike V.Y., Yarandi N., Petraro P., Ayettey R.G., Treu J.A., Katz D.L. (2016). Inclusion of walnut in the diets of adults at risk for type 2 diabetes and their dietary pattern changes: A randomized, controlled, cross-over trial. BMJ Open Diabetes Res. Care.

[41] Neale E.P., Tapsell L.C., Martin A., Batterham M.J., Wibisono C., Probst Y.C. (2017). Impact of providing walnut samples in a lifestyle intervention for weight loss: A secondary analysis of the HealthTrack trial. Food Nutr. Res..

[42] Bitok E., Jaceldo-Siegl K., Rajaram S., Serra-Mir M., Roth I., Feitas-Simoes T., Ros E., Sabate J. (2017). Favourable nutrient intake and displacement with long-term walnut supplementation among elderly: Results of a randomised trial. Br. J. Nutr..

[43] Jaceldo-Siegl K., Sabate J., Rajaram S., Fraser G.E. (2004). Long-term almond supplementation without advice on food replacement induces favourable nutrient modifications to the habitual diets of free-living individuals. Br. J. Nutr..

[44] Pearson K.R., Tey S.L., Gray A.R., Chisholm A., Brown R.C. (2017). Energy compensation and nutrient displacement following regular consumption of hazelnuts and other energy-dense snack foods in non-obese individuals. Eur. J. Nutr..

[45] Lafreniere J., Couillard C., Lamarche B., Laramee C., Vohl M.C., Lemieux S. (2019). Associations between self-reported vegetable and fruit intake assessed with a new web-based 24-h dietary recall and serum carotenoids in free-living adults: A relative validation study. J. Nutr. Sci..

